# Association between Life's Essential 8 and frailty among the United States older people

**DOI:** 10.3389/fpubh.2025.1554687

**Published:** 2025-07-16

**Authors:** Na Zhao, Yameng Fan

**Affiliations:** ^1^Office for Nosocomial Infection Control, Xi'an Central Hospital, Xi'an, China; ^2^School of Public Health, Xi'an Medical University, Xi'an, China

**Keywords:** Life's Essential 8, frailty, older people, CVH, NHANES

## Abstract

**Background:**

The association between cardiovascular disease health (CVH) and frailty has not been conclusive. The American Heart Association (AHA) has proposed the Life's Essential 8 (LE8) score as an indicator of CVH. We sought to examine the association between LE8 and frailty among older people from the US general population.

**Methods:**

We analyzed data from the 2015–2018 National Health and Nutrition Examination Survey and included older people aged ≥60 years. The LE8 score includes 8 metrics (4 health behaviors and 4 health factors). Frailty status was assessed using the FRAIL scale based on 5 criteria. Multivariate logistic regression analyses were used to assess associations.

**Results:**

A total of 2,511 older people (aged 60 years, with a weighted number of 49,532,259) were included. Among them, 1,294 (weighted percentage: 46.0%) were male and 1,217 (weighted percentage: 54.0%) were female. Older people with a higher LE8 score had a lower risk of frailty, the odds ratio (OR) for each standard deviation (SD) increase in the LE8 score was 0.59 (95% CI, 0.48–0.71, *P* < 0.001). Similar results were observed in the associations of the health behaviors [OR 0.62 (95% CI, 0.50–0.78), *P* < 0.001] and health factors [OR 0.76 (95% CI, 0.60–0.96), *P* = 0.024] with frailty. After excluding older people with poor health status, the results remained significant, the OR for per SD score increase was 0.57 (95% CI, 0.46–0.69, *P* < 0.001).

**Conclusion:**

A higher LE8 score was associated with lower risk of frailty among older people in the US. Adherence to optimal CVH scores may be beneficial in helping prevent frailty.

## Introduction

Globally, the aging population is increasing much faster than in the past, and this demographic transition will have a serious impact on all aspects of society. According to the World Health Organization, the proportion of the world's population over 60 years will nearly double from 12% to 22% between 2015 and 2050 (1). Frailty, a complex age-related clinical condition, is characterized by a decline in physiological function and reserves across multiple systems, including fatigue, resistance, ambulation, illness, and weight loss, especially in the context of aging (2–4). The prevalence of frailty among individuals aged 65 years and older has increased by 5.1% in the past decade, underscoring the importance of early intervention (5, 6). Due to aging and frailty, countries worldwide will face increased pressure on health care systems and a huge economic burden (7). Given the poor health outcomes related to frailty, it is imperative to identify modifiable risk factors to delay or prevent the development of frailty.

Despite the decline in mortality rates from cardiovascular disease (CVD) over the years, CVD remains a major chronic disease worldwide and a leading cause of death (8). While CVD prevalence in the general population was 9.04%, it is significantly higher among older adults, where it reaches 16.85% (9), and even higher in those with cancer, diabetes, hypertension, and other diseases (10–13). Based on such findings, the American Heart Association (AHA) updated the Life's Essential 8 (LE8) score as a measure of cardiovascular health (CVH) to reduce the prevalence of CVD and further improve the health of the general population. The components of LE8 include healthy diet, physical activity, nicotine exposure, sleep, body mass index, blood lipids, blood glucose, and blood pressure, which are closely related to aging (8). For example, the promotion of physical activity in older adults goes beyond its well-documented benefits on physical, psychological, and cognitive health (14); Strength training emerges as a key strategy for healthy aging, as it not only prevents frailty and falls but also improves the quality of life in older adults (15).

It has been reported that if all US adults maintained a high CVH, 2.0 million CVD events would be prevented each year (16). Recent studies have shown that having better CVH is associated with more favorable long-term health outcomes for older people, especially in terms of cognitive function and quality of life (17–19). Thus, improving CHV may be an appropriate prevention and management strategy to reduce the prevalence of frailty.

Given the evidence that better cardiovascular health is associated with improved outcomes in older adults, this study aimed to analyze the association between the LE8 score and frailty in a large, nationally representative, and ethnically diverse sample of older adults in the US, using data from the NHANES 2015–2018.

## Methods

### Study participants

**Figure 1:**
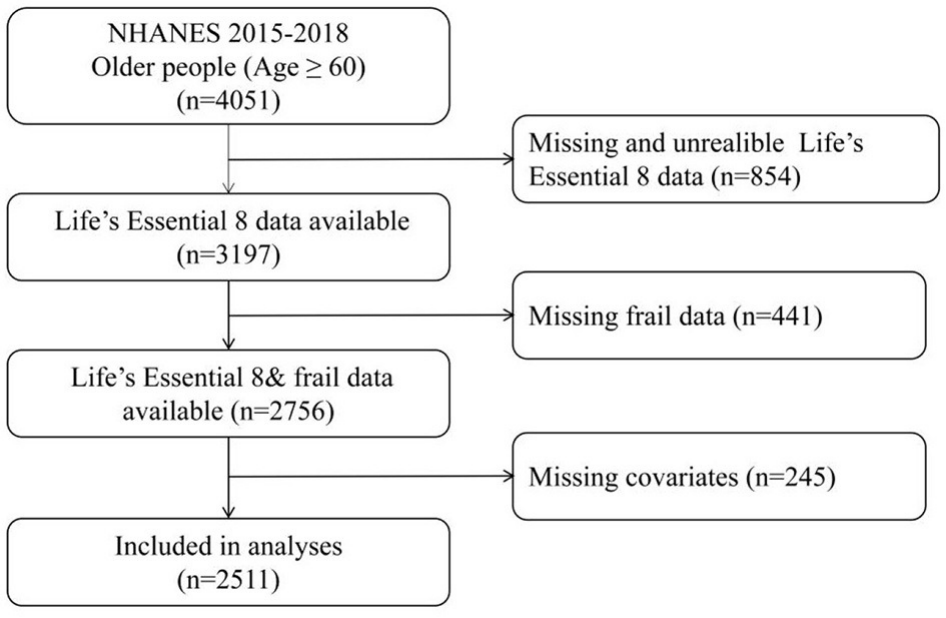
Flow diagram of the study sample selection.

Data were extracted from the National Health and Nutrition Examination Survey (NHANES), which conducts continuous 2-year cycle cross-sectional surveys. These surveys are administered by the National Center for Health Statistics (NCHS) under the Centers for Disease Control and Prevention (CDC) (20). All procedures performed in the study were in accordance with recommendations of the CDC and the NCHS. Informed consent was obtained from all individual participants. The present analysis combined data from the last two NHANES cycles (NHANES 2015–2016 and 2017–2018). We included a total of 4,051 individuals aged ≥60 years. Due to missing data being unable to provide reliable results, it is necessary to delete them. Exclusion criteria were as follows: 854 participants had missing data for any component of the LE8 metrics, 441 had missing data for frailty components, and 245 had missing covariate data. After exclusions, 2,511 individuals were included in the final analysis (Figure 1).

### Development of LE8

The LE8 score is a standardized tool defined by the AHA to measure and monitor CVH in individuals and populations. The LE8 includes eight metrics and has been grouped into 2 domains of health behaviors (healthy diet, physical activity, nicotine exposure, sleep) and health factors (body mass index, blood lipids, blood glucose, blood pressure). The PA score, the nicotine exposure score and the sleep score were obtained according to a self-reported questionnaire. The BMI score, blood lipids score, blood glucose score, and BP score were obtained according to examination data and laboratory data. The specific scoring standards for each metric are shown in Supplementary Table 1. In brief, each of the 8 CHV metrics was scored with a range of 0–100 points. The LE8 score was calculated as the unweighted average of all 8-component metrics. Furthermore, the LE8 score was grouped into 3 categories, high CVH (80–100 points), moderate CVH (50–79 points), low CVH (0–49 points) by the AHA (8). The health behaviors and the health factors were also grouped into three categories of high, moderate, and low. Specifically, diet information was evaluated by the Healthy Eating Index 2015 (HEI-2015) in this study, which is a quality index developed in partnership by USDA and NCI, researchers. In summary, higher HEI-2015 scores reflect better diet quality (21, 22). The components and scoring standards of HEI-2015 are shown in Supplementary Table 2.

### Assessment of physical frailty status

Frailty status was assessed using the FRAIL scale, which consists of five simple questions, each requiring a yes or no answer, with a score of one given to each “yes” response. Participants meeting three or more items were considered as frail; those who met one or two items were pre-frail; and those who met none of the items were robust. The FRAIL scale is entirely based on self-report with the following criteria (23–25):

1. Fatigue: defined as “yes” of these responses “more than half of the days” or “nearly every day” to the question “How often have you been feeling tired or having little energy?” yes = 1, no = 0
2. Resistance: defined as “yes” of these responses “some difficulty”, “much difficulty”, “unable to do” or “do not do this activity” to the question “How much difficult do you have walking up ten stairs by yourself ?” yes = 1, no = 0
3. Ambulation: defined as “yes” of these responses “some difficulty”, “much difficulty”, “unable to do” or “do not do this activity” to the question “How much difficult do you have walking for a quarter of a mile alone and without using special equipment ?” yes = 1, no = 0
4. Illness: defined as “yes” of participants with 5 or more of a total of 11 diseases (hypertension, diabetes, cancer, chronic lung disease, heart attack, congestive heart failure, angina, asthma, arthritis, stroke, and kidney disease). yes = 1, no = 0
5. Weight loss: defined as “yes” of weight loss by a decrease of ≥5% within the last 12 months, the percentage of weight change is calculated as: [(weight 1 year ago – current weight)/weight 1 year ago] × 100. yes = 1, no = 0

### Covariates

Covariates (age, gender, race, marital status, education, income) were included in this study. Age was stratified into 2 strata: 60–69 years, 70 years or older. gender was classified as male or female. Race was classified as Mexican American, other Hispanic, Non-Hispanic White, non-Hispanic Black, and Other. The educational level was classified as lower than high school, high school, and higher than high school. Marital status was categorized as single or separated (widowed, divorced, separated, never married), and coupled (married, living with partner). Income was expressed using the monthly poverty level index and was classified into 3 groups ( ≤ 1.30, 1.31–1.85, >1.85), with a higher index indicating better income (26). All demographic characteristics were collected through questionnaire interviews. The general health condition of the participants was collected using self-report questionnaires and was classified as excellent, very good, good, fair, and poor.

### Statistical analysis

The NHANES uses a complex and multistage probability sampling design; thus all analyses accounted for sample weights in this study. Categorical variables were described as frequencies and weighted percentages, and continuous variables were described as weighted means and standard error (SE). The baseline characteristics were described according to the frail and non-frail group. The baseline characteristics were compared using the Chi-square test for categorical variables and the unadjusted linear regressions for continuous variables.

The odds of being frail compared to non-frail (the combined pre-frail or robust groups) were explored using weighted binary logistic regression, after adjusting for possible confounders. Model 1 was unadjusted; Model 2 was adjusted for age, gender, and race; Model 3 was further adjusted for marital status, education, and the poverty level index. Potential linear or nonlinear relationships were examined with restricted cubic splines via the SAS macro “%RCS_Reg” (27). Then, the multinomial logistic regression model was used to estimate the odds of three frailty categories (pre-frail vs. robust; frail vs. robust). Subgroup analyses were conducted to test according to age strata, gender, race, marital status, education, and income. Finally, Sensitivity analyses were conducted by removing people with cancer or stroke to evaluate the robustness of our results. Data management and restricted cubic splines were performed using SAS v.9.4 (SAS Institute, Cary, North Carolina, USA) and all statistical analyses were performed using Stata v.17.0 (IBM, Armonk, NY, USA). The two-tailed *P* < 0.05 was considered statistically significant.

## Results

### Characteristics of the study population

The characteristics of the study population were described dividing patients into the frail and non-frail group in Table 1. A total of 2,511 (weighted number, 49,532,259) older participants aged ≥60 years were included, with 1,376 [weighted percentage (WP), 57.9%] participants aged 60–69 years and 1,135 (WP, 42.1%) aged ≥70 years; 1,294 (WP, 46.0%) were men and 1,217 (WP, 54.0%) were women. There were significant differences in terms of age, gender, education, or family monthly poverty level in the frail and non-frail groups, while no significant differences were observed in race and marital status between the two groups. In summary, older, female, low-education, and low-income participants are more likely to be frail.

**Table 1:** Characteristics of the participants and CVH metrics by frail status, NHANES 2015–2018.

**Variables**	**Overall (*n* = 2,511)**	**Non-frail (*n* = 2,321)**	**Frail (*n* = 190)**	***P*^b^ value**
Weighted number	49,532,259	46,228,093	3,304,165	
**Age strata**, ***n*** **(%)**				0.002
60–69	1,376 (57.9)	1,296 (59.2)	80 (39.3)	
≥70	1,135 (42.1)	1,025 (40.8)	110 (60.7)	
**Gender**, ***n*** **(%)**				0.001
Male	1,294 (46.0)	1,217 (47.0)	77 (32.3)	
Female	1,217 (54.0)	1,104 (53.0)	113 (67.7)	
**Race/Ethnicity**, ***n*** **(%)**				0.253
Mexican American	330 (4.0)	317 (4.1)	13 (2.3)	
Other Hispanic	295 (3.8)	276 (3.7)	19 (3.4)	
Non-Hispanic White	1,098 (78.6)	996 (78.9)	102 (76.1)	
Non-Hispanic Black	495 (7.2)	459 (7.1)	36 (8.6)	
Other	293 (6.4)	273 (6.1)	20 (9.6)	
**Education**, ***n*** **(%)**				<0.001
Less than High school	576 (11.2)	530 (10.8)	46 (15.9)	
High school	1,334 (55.6)	1,213 (54.5)	121 (71.7)	
More than high school	601 (33.2)	578 (34.7)	23 (12.4)	
**Marital status**, ***n*** **(%)**				0.121
Coupled	1,523 (66.6)	1,421 (67.1)	102 (58.9)	
Single or separated	988 (33.4)	900 (32.9)	88 (41.1)	
**Family monthly poverty level**, ***n*** **(%)**				<0.001
≤ 1.30	706 (15.1)	637 (14.3)	69 (26.9)	
1.31–1.85	452 (11.8)	404 (11.2)	48 (20.5)	
>1.85	1,353 (73.1)	1,280 (74.5)	73 (52.6)	
**Life's Essential 8 score, mean (SE)**				
Diet score (HEI 2015 score)	39.3 (1.1)	39.7 (1.2)	32.6 (2.3)	0.011
Physical activity score	64.9 (1.7)	66.0 (1.8)	48.6 (5.0)	0.004
Nicotine exposure score	77.7 (0.8)	78.4 (0.9)	68.2 (4.9)	0.060
Sleep health score	86.7 (0.7)	87.2 (0.7)	79.5 (2.5)	0.005
Body mass index score	57.1 (1.2)	58.0 (1.3)	44.0 (2.9)	<0.001
Blood lipids score	62.0 (0.8)	61.8 (0.9)	65.9 (2.0)	0.075
Blood glucose score	66.9 (1.2)	67.5 (1.2)	58.8 (2.3)	0.001
Blood pressure score	50.1 (1.2)	50.5 (1.3)	44.9 (3.8)	0.196
LE 8 score	63.1 (0.6)	63.7 (0.7)	55.3 (1.0)	<0.001
**Cardiovascular health**^a^, ***n*** **(%)**				<0.001
Low CVH	551 (17.5)	478 (16.3)	73 (33.0)	
Moderate CVH	1779 (70.9)	1667 (71.5)	112 (63.7)	
High CVH	181 (11.6)	176 (12.2)	5 (3.3)	
**Health behaviors**, ***n*** **(%)**				<0.001
Low	585 (18.7)	518 (17.7)	67 (32.2)	
Moderate	1288 (49.8)	1189 (49.5)	99 (53.4)	
High	638 (31.5)	614 (32.8)	24 (14.4)	
**Health factors**, ***n*** **(%)**				0.043
Low	889 (30.4)	791 (29.7)	98 (41.2)	
Moderate	1,388 (56.2)	1,306 (56.5)	82 (51.6)	
High	234 (13.4)	224 (13.8)	10 (7.2)	

All values were weighted. LE8, Life's Essential 8 score; HEI, healthy eating index; CVH, cardiovascular health; SE, standard error.

^a^Low CVH was defined as a LE8 score of 0–49, moderate CVH of 50–79 and high CVH of 80–100.

^b^*P*-values were calculated by Chi-square tests for categorical variables and unadjusted linear regressions was used for continuous variables.

The weighted mean of the LE8 score of the study participants was 63.1 (95% CI, 61.8–64.4). The LE8 score was significantly higher in the non-frail group than in the frail group (*P* < 0.001). Compared to the frail group, non-frail participants had a higher diet score, PA score, nicotine exposure score, sleep health score, body mass index score, and blood glucose score. However, there was no significant difference in nicotine exposure score, blood lipid score, and blood pressure score between the two groups. Participants with low, moderate, and high CVH were 551 (WP, 17.5%), 1,779 (WP, 70.9%), and 181 (WP, 11.6%), separately. LE8 has been grouped into health behaviors and health factors by AHA. Either health behaviors (*P* < 0.001) or health factors (*P* = 0.043) showed significant differences in the frail and non-frail groups.

### LE8 score and frailty

Table 2 presents the results of the weighted multiple logistic regression analysis. In the unadjusted analysis Model 1, compared with the low CVH group, the odds ratios (OR) for frailty were 0.44 (95% CI, 0.28–0.69; *P* = 0.001) in the moderate CVH group and 0.14 (95% CI, 0.04–0.50; *P* = 0.004) in the high CVH group. In further adjusted Model 2, the high CVH group was also associated with lower odds of frailty (OR: 0.13; 95% CI, 0.04–0.47; *P* = 0.003), compared to the low CVH group. After additionally adjusting for education, marital status, and family monthly poverty level in Model 3, the association of the LE8 score with frailty (OR: 0.21; 95% CI, 0.07–0.67; *P* = 0.010) attenuated but remained significant. In all three models, a significantly lower odds of frailty was observed in participants with a higher LE8 score. The ORs of frailty in Models 1, 2, and 3 were 0.56 (95% CI, 0.46–0.68; *P* < 0.001), 0.52 (95% CI, 0.43–0.64; *P* < 0.001), and 0.59 (95% CI, 0.48–0.71; *P* < 0.001) for each SD increase of the LE8 score, respectively.

**Table 2:** Odds ratios and 95% confidence intervals of Life's Essential 8 scores for two frailty status (frail vs. non-frail, *n* = 2,511).

**LE 8 score**	**Model 1** ^**a**^		**Model 2** ^**b**^		**Model 3** ^**c**^	
	**OR (95% CI)**	***P***	**OR (95% CI)**	***P***	**OR (95% CI)**	***P***
Low	1 (Reference)	/	1 (Reference)	/	1 (Reference)	/
Moderate	0.44 (0.28–0.69)	0.001	0.40 (0.26–0.63)	<0.001	0.49 (0.32–0.75)	0.002
High	0.14 (0.04–0.50)	0.004	0.13 (0.04–0.47)	0.003	0.21 (0.07–0.67)	0.010
Per SD increase	0.56 (0.46–0.68)	<0.001	0.52 (0.43–0.64)	<0.001	0.59 (0.48–0.71)	<0.001

OR, odds ratio; CI, confidence interval.

^a^No covariates were adjusted.

^b^Age, gender, and race were adjusted.

^c^Age, gender, race, education level, rate of family income to poverty, marital status were adjusted.

**Figure 2:**
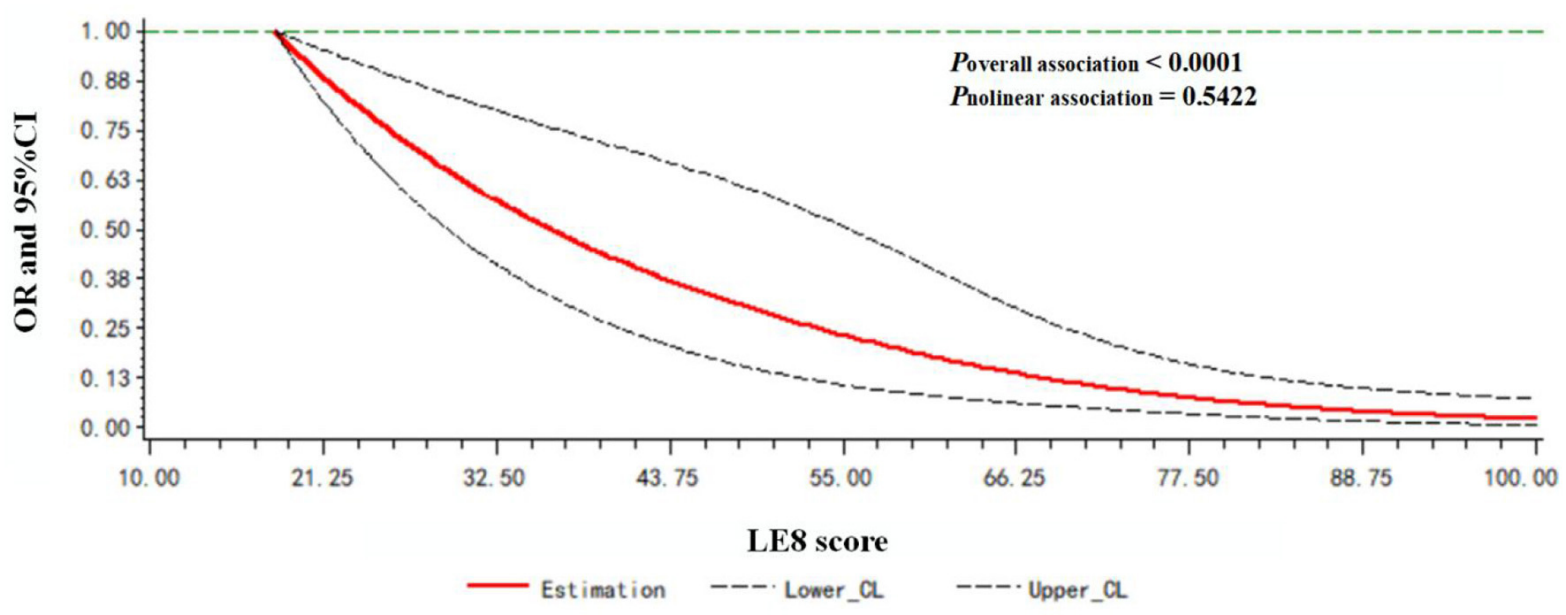
Dose-response relationship between the LE8 score and frailty status. Models were adjusted for age, gender, race, education level, rate of family income to poverty, and marital status.

A dose-response relationship between the LE8 score and frailty status (frail vs. non-frail) was observed. As shown in Figure 2, the LE8 score was negatively associated with frailty in a linear manner (*P*_overallassociation_ <0.0001; *P*_nonlinearassociation_ = 0.5422).

The odds of being pre-frail or frail compared to robust were further explored using multinomial logistic regression. After full adjustment, participants in the high CVH group were less likely to be pre-frail (*P* < 0.001) or frail (*P* < 0.001) compared with participants in the low CVH group (Table 3).

**Table 3:** Odds ratios and 95% confidence intervals of Life's Essential 8 scores for three frailty status (pre-frail vs. robust/frail vs. robust, *n* = 2511).

**LE 8 score**	**Pre-frail vs. robust**		**Frail vs. robust**	
	**OR (95% CI)**	***P***	**OR (95% CI)**	***P***
Low	1 (Reference)	/	1 (Reference)	/
Moderate	0.66 (0.45, 0.97)	0.004	0.40 (0.24, 0.67)	0.001
High	0.39 (0.21, 0.72)	<0.001	0.14 (0.05, 0.44)	<0.001
Per SD increase	0.77 (0.65, 0.91)	<0.001	0.52 (0.42, 0.64)	<0.001

OR, odds ratio; CI, confidence interval.

Adjusted for age, gender, race, education level, rate of family income to poverty, marital status.

### Health behaviors/health factors and frailty

We also analyzed the associations of the health behavior/health factors scores with frailty by logistic regression analysis in Table 4. In the fully adjusted Model 3, compared with low health behaviors group, the ORs for frailty were 0.64 (95% CI, 0.40–1.03; *P* = 0.063) in the moderate behaviors group and 0.32 (95% CI, 0.15–0.68; *P* = 0.004) in the high health behaviors group, respectively. When per SD increase in the health behaviors score, the OR for frailty was 0.58 (95% CI, 0.47–0.72; *P* < 0.001) in Model 1, 0.55 (95% CI, 0.44–0.69; *P* < 0.001) in Model 2, and 0.62 (95% CI, 0.50–0.78; *P* < 0.001) in Model 3. For all models, a high healthy behaviors score was significantly associated with lower odds of frailty.

**Table 4:** Odds ratios and 95% confidence intervals of Life's Essential 8 scores for frailty status (*n* = 2,511).

**LE 8**	**Model 1** ^**a**^		**Model 2** ^**b**^		**Model 3** ^**c**^	
	**OR (95% CI)**	***P***	**OR (95% CI)**	***P***	**OR (95% CI)**	***P***
**Health behaviors score**						
Low	1 (Reference)	/	1 (Reference)	/	1 (Reference)	/
Moderate	0.59 (0.37–0.96)	0.034	0.55 (0.34–0.89)	0.016	0.64 (0.40–1.03)	0.063
High	0.24 (0.11–0.53)	0.001	0.23 (0.10–0.50)	0.001	0.32 (0.15–0.68)	0.004
Per SD increase	0.58 (0.47–0.72)	<0.001	0.55 (0.44–0.69)	<0.001	0.62 (0.50–0.78)	<0.001
**Health factors score**						
Low	1 (Reference)	/	1 (Reference)	/	1 (Reference)	/
Moderate	0.66 (0.44–0.98)	0.041	0.62 (0.42–0.93)	0.022	0.68 (0.47–1.00)	0.053
High	0.38 (0.14–1.04)	0.059	0.39 (0.14–1.09)	0.071	0.50 (0.18–1.34)	0.163
Per SD increase	0.71 (0.57–0.89)	0.004	0.70 (0.56–0.88)	0.004	0.76 (0.60–0.96)	0.024

OR, odds ratio; SD, standard deviation; CI, confidence interval.

^a^No covariates were adjusted.

^b^Age, gender, and race were adjusted.

^c^Age, gender, race, education level, rate of family income to poverty, marital status were adjusted.

Compared with the low health factors group, the ORs for frailty were 0.68 (95% CI, 0.47–1.00; *P* = 0.053) in the moderate group and 0.50 (95% CI, 0.18–1.34; *P* = 0.163) in the high group after full adjustment. For the per SD increase in health factors score, the ORs for frailty were 0.71(95% CI, 0.57–0.89; *P* = 0.004) in Model 1, 0.70 (95% CI, 0.56–0.88; *P* = 0.004) in Model 2, and 0.76 (95% CI, 0.60–0.96; *P* = 0.024) in Model 3.

**Figure 3:**
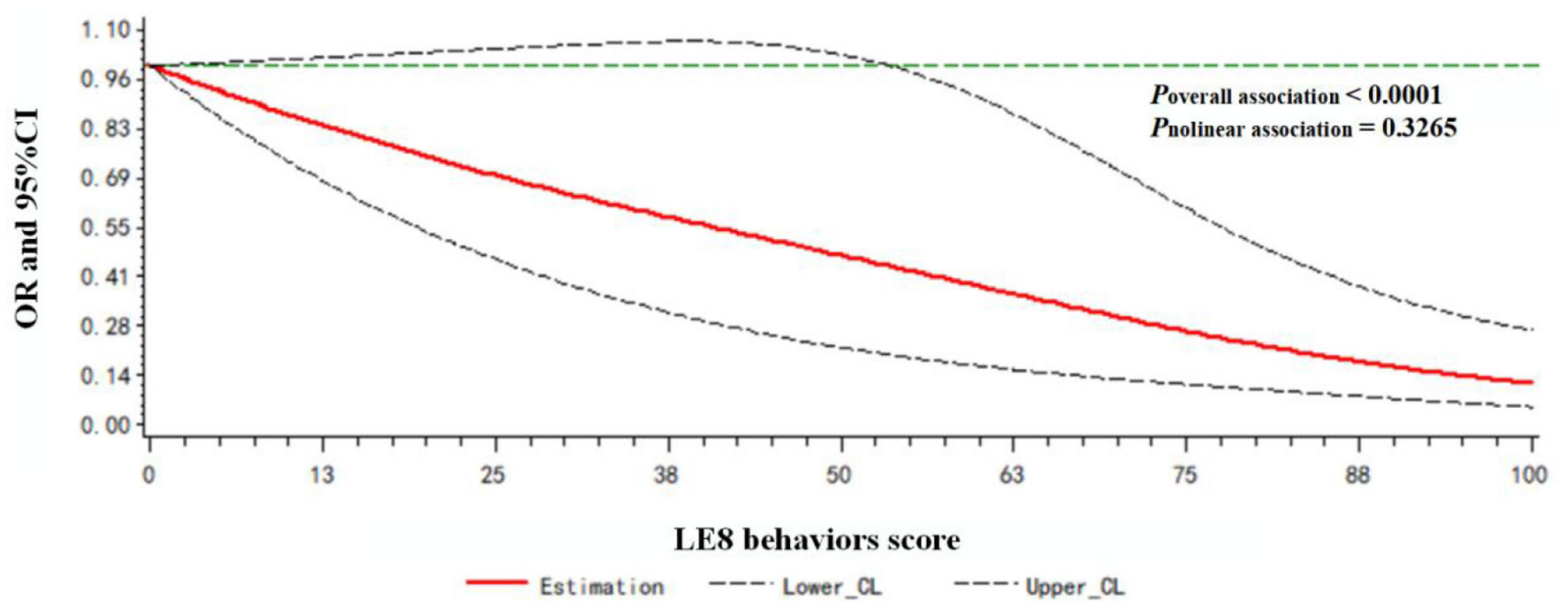
Dose-response relationship between the LE8 behaviors score and frailty status. Models were adjusted for age, gender, race, education level, rate of family income to poverty, and marital status.

**Figure 4:**
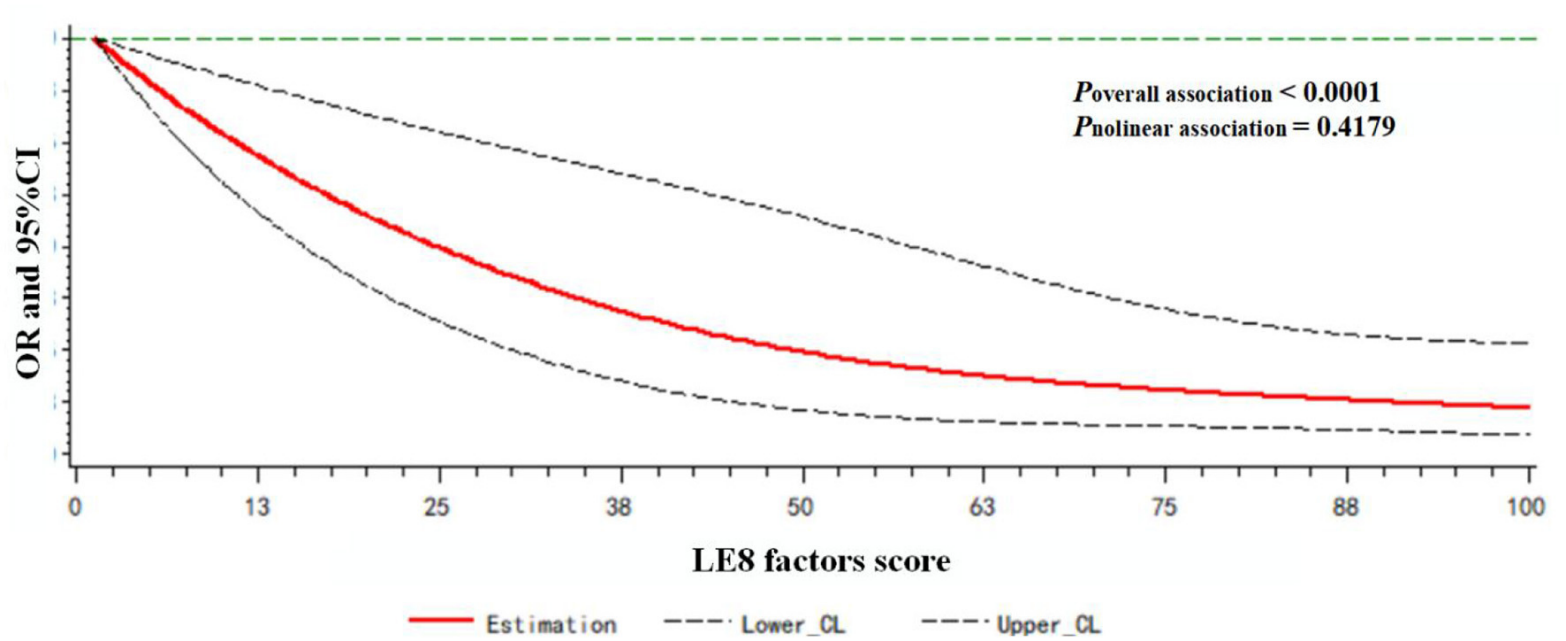
Dose-response relationship between the LE8 factors score and frailty status. Models were adjusted for age, gender, race, education level, rate of family income to poverty, and marital status.

A dose-response relationship between the LE8 behaviors score/LE8 factors score and frailty status (frail vs. non-frail) was observed. As shown in Figure 3, the LE8 behaviors score was negatively associated with frailty in a linear manner (*P*_overallassociation_ <0.0001; *P*_nonlinearassociation_ = 0.3265). Similarly, the LE8 factors score was negatively associated with frailty in a linear manner (*P*_overallassociation_ <0.0001; *P*_nonlinearassociation_ = 0.4179), as presented in Figure 4.

### Subgroup analysis

**Figure 5:**
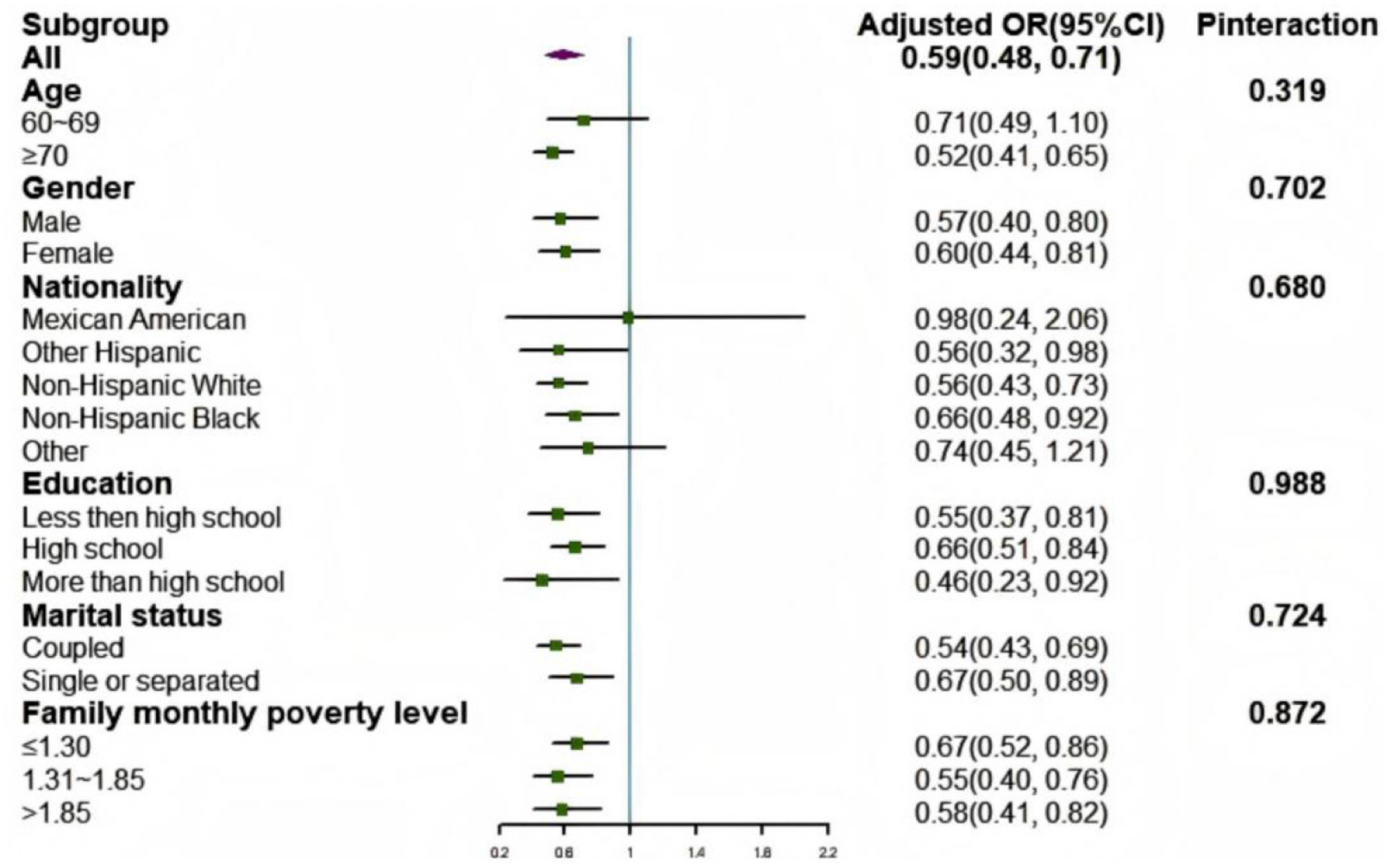
Subgroup analysis of the association between LE8 scores and frailty status. Each category was adjusted for age, gender, race, education level, rate of family income to poverty, and marital status. OR, odds radio; CI, confidence interval.

In the subgroup analysis, no significant interaction effects were found between the LE8 score and all covariates, including age, gender, nationality, education, marital status, and family monthly poverty level, with frailty in the fully adjusted model (*P*_interaction_ > 0.05; Figure 5).

### Sensitivity analyses

The association between the LE8 score and frailty did not appreciably change in the sensitivity analyses after excluding participants who suffered from cancer (*n* = 1,987; Supplementary Table 3) or stroke (*n* = 2,349; Supplementary Table 4).

## Discussion

In this cross-sectional study, our findings indicated that higher levels of CHV assessed by the LE8 score were significantly associated with a lower risk of frailty in older people in the US. After excluding participants with cancer or stroke, the results still remained significant.

In our study, we observed that the prevalence of frailty was 7.57% in older residents of the US, which was lower than that observed for older participants living in the community setting (10.7%) reported by a systematic review (28). One possible explanation is that the prevalence of frailty varies enormously, especially in different countries and different ethnic populations. Secondly, due to the lack of standardized assessments, there are great differences in the prevalence of frailty. In this study, we used the FRAIL scale to assess the frailty status rather than other alternative frailty assessments, such as Frailty Phenotype or Frailty Index. As these assessments share overlapping metrics with LE8, which could potentially introduce spurious associations between LE8 and frailty. Furthermore, our study indicated that women, single, and people with lower education and income had higher prevalence for frailty, which was in agreement with previous studies (7, 29, 30).

The definition of CVH was based on 4 health behaviors and 4 health factors. A better CVH was associated with better CVD-free survival, longer total longevity, and a higher quality of life (8). Previous studies on associations between CVH metrics and frailty have shown inconsistent results. As a modifiable factor, most studies have indicated that diet plays an important role in older individuals. A better diet is required to provide adequate energy and nutrition to help prevent or delay frailty. Gimeno et al. (31) showed that an optimal diet can prevent frailt. Fan et al. (26) observed that a higher HEI-2015 was inversely associated with lower odds of physical frailty among older individuals. However, no association was observed between a high Mediterranean Diet Score (MDS) and incident frailty in a prospective cohort study from Hong Kong (32). Most studies on the associations between physical activity and frailty have reached an identical conclusion. Physical activity is considered one of the main strategies to counteract physical impairment related to frailty (33). A randomized controlled trial found that a multicomponent exercise program improves physical performance and delays frailty in older people (34). Regarding BMI, most studies consider obesity or emaciation as risk factors for frailty (35–37). Furthermore, both obesity and large waist circumference were associated with systemic inflammation and insulin resistance, which was also shown to be associated with frailty (38). The sleep health index, as a new CVH metric added by the AHA, is also an influencing factor for the appearance of frailty. However, the potential mechanisms affecting how sleep quality acts on frailty status are unknown. According to previous studies, sleep quality was well established to have an impact on the development of frailty (39–41). A meta-analysis including 41,233 individuals revealed that longer and shorter durations of sleep increases the risk of frailty (42). In addition, previous studies on the relationship between blood pressure and blood sugar with frailty had inconsistent results. Wang et al. found there was no association between frailty and biometric factors, such as blood pressure, blood lipids, and blood glucose, in a prospective study by the UK Biobank (43). However, in a cross-sectional study in Brazil, frail adults had higher BP, lower HDL and more abdominal fat than non-frail adults (44).

The reasons for these inconsistencies remain the subject of further research. A potential explanation for this discrepancy lies in differences in population and assessment methods. Furthermore, these inconsistencies might also reflect the complex process of frailty. Frailty is likely the result of a multiple of CVH metrics working together rather than a single CVH metric. Therefore, we should use an aggregative indicator such as LE8 rather than a single metric to assess the association between CVH and frailty.

Several previous studies have evaluated the association between Simple 7 (LS7) and frailty. A study by Irving Medical Center of Columbia University found that a higher LS7 score was associated with a lower prevalence of frailty in later life (45). In Spain, a prospective cohort study revealed that an ideal LS7 was associated with a reduced risk of frailty in older people (46). A study by the UK Biobank observed that a higher LS7 score in midlife was associated with a lower risk of physical frailty in later life (43). However, LS7 may not reflect the full scope of health behaviors and practices in the current context. After the updated and improved approach is applied to measuring, monitoring, and modifying CVH, LE8 can better represent the full range of CVH compared to LS7. Our study evaluated the relationship between LE8 and frailty and observed that higher LE8 was significantly associated with a lower risk of frailty in older people from the USA. Compared with health factors, health behavior is easier to change and achieves obvious results to prevent frailty. According to existing research, frailty is difficult to reverse and does not have a specific treatment. Therefore, strategies to prevent and slow the progression of frailty are essential. Our findings indicated that a more rigorous standard of cardiovascular health, especially healthy behaviors, might be preferable to reduce frailty. In addition, no significant interaction effects were found in this study, which revealed that the development of CHV to improve the LE8 score was effective to reduce the incidence of frailty in all populations.

Notably, no significant interaction effects were observed in our study, indicating that the relationship between the LE8 score and frailty was consistent across age, gender, nationality, education, marital status, and family monthly poverty level. Similarly, among cancer survivors, there were no significant interaction effects stratified by factors including gender, race, education level, and marital status (47). This consistency highlights that the LE8 score is a robust indicator for assessing frailty risk. It remains reliable and unaffected by diverse demographic factors.

The present study has several strengths. To our knowledge, this is the first study to explore the association between LE8 and frailty in the general population of older individuals in the US. Second, most previous studies have been restricted to single factors, not an aggregative indicator. As a comprehensive index, LE8 can explore the relationship with frailty more comprehensively. Finally, we used data from NHANES, a large nationally representative data, which ensures the generalizability of our findings. However, several limitations should also be noted in the present study. First, as a cross-sectional study design, we were unable to establish a causal relationship between frailty and the LE8 score. Due to a potential bidirectional relationships between LE8 and frailty, we should not exclude the effect of frailty on LE8. This emphasized the need for longitudinal research in the future. Second, although the FRAIL scale is widely used, it is entirely based on self-reported data, which may introduce bias and cannot reflect the actual frailty status. Lastly, despite our meticulous efforts to control for a broad range of potential confounders, it is crucial to acknowledge that unmeasured variables may still influence our results.

## Conclusions

In this nationally representative sample of older people from the US, we observed an inverse association between LE8 and frailty. This research emphasizes the vital role of CVH in reducing frailty among older people, indicating adherence to optimal CVH levels may translate into the prevention of frailty and the reduction of public health burdens. Given the rational findings and several limitations in the present study, the results should be further validated in the large prospective cohort study.

## Data Availability

Publicly available datasets were analyzed in this study. This data can be found here: https://wwwn.cdc.gov/nchs/nhanes.
