# Supplementary material for: Association between Life's Essential 8 and frailty among the United States older people
Source: Front Public Health. 2025 Jul 16;13:1554687. doi: 10.3389/fpubh.2025.1554687 (PMC12307139; doi:10.3389/fpubh.2025.1554687)
Supplement: Supplementary file 1 [file Table_1.DOCX]

**Supplementary material**

**Association between Life’s Essential 8 and frailty among the United States older people**

**Table of Contents**

**Additional Table 1............................................................................................................................................................1**

**Additional Table 2............................................................................................................................................................5**

**Additional Table 3............................................................................................................................................................6**

**Additional Table 4............................................................................................................................................................7**

**Additional Table 1. Metrics for Measurement and Quantitative Assessment of CVH**

| Domain | CVH metric | Method of measurement | Quantification of CVH metric: adults  (≥20 y of age) |
| --- | --- | --- | --- |
| Health behaviors | Diet | Self-reported daily intake of a DASH-style eating pattern | Quantiles of DASH-style diet adherence or HEI-2015 (population)  Scoring (population):  Points Quantile  100 ≥95th percentile (top/ideal diet)  80 75th–94th percentile  50 50th–74th percentile  25 25th–49th percentile  0 1st–24th percentile (bottom/least ideal quartile) |
|  | PA | Self-reported minutes of moderate or vigorous PA per week | Metric: Minutes of moderate- (or greater) intensity activity per week  Scoring:  Points Minutes  100 ≥150  90 120–149  80 90–119  60 60–89  40 30–59  20 1–29  0 0 |
|  | Nicotine  exposure | Self-reported use of cigarettes or inhaled NDS | Metric: Combustible tobacco use or inhaled NDS use; or secondhand smoke exposure  Scoring:  Points Status  100 Never smoker  75 Former smoker, quit ≥5 y  50 Former smoker, quit 1–<5 y  25 Former smoker, quit <1 y, or currently using inhaled NDS  0 Current smoker  Subtract 20 points (unless score is 0) for living with active indoor smoker in home |
|  | Sleep health | Self-reported average hours of sleep per night | Metric: Average hours of sleep per night  Scoring:  Points Level  100 7–<9  90 9–<10  70 6–<7  40 5–<6 or ≥10  20 4–<5  0 <4 |
| Health factors | BMI | Body weight (kilograms) divided by height squared (meters squared) | Metric: BMI (kg/m2)  Scoring:  Points Level  100 <25  70 25.0–29.9  30 30.0–34.9  15 35.0–39.9  0 ≥40.0 |
|  | Blood lipids | Plasma total and HDL cholesterol with calculation of non–HDL cholesterol | Metric: Non–HDL cholesterol (mg/dL)  Scoring:  Points Level  100 <130  60 130–159  40 160–189  20 190–219  0 ≥220  If drug-treated level, subtract 20 points |
|  | Blood glucose | Measurement: FBG or casual HbA1c | Metric: FBG (mg/dL) or HbA1c (%)  Scoring:  Points Level  100 No history of diabetes and FBG <100 (or HbA1c<5.7)  60 No diabetes and FBG 100–125 (or HbA1c 5.7–6.4)  40 Diabetes with HbA1c <7.0  30 Diabetes with HbA1c 7.0–7.9  20 Diabetes with HbA1c 8.0–8.9  10 Diabetes with HbA1c 9.0–9.9  0 Diabetes with HbA1c ≥10.0 |
|  | BP | Measurement: Appropriately measured systolic and diastolic BPs | Metric: Systolic and diastolic BPs (mmHg)  Scoring:  Points Level  100 <120/<80 (optimal)  75 120–129/<80 (elevated)  50 130–139 or 80–89 (stage 1 hypertension)  25 140–159 or 90–99  0 ≥160 or ≥100  Subtract 20 points if treated level |

1. Lloyd-Jones DM, Allen NB, Anderson CAM, et al. Life's Essential 8: Updating and Enhancing the American Heart Association's Construct of Cardiovascular Health: A Presidential Advisory From the American Heart Association. *Circulation*. Aug 2 2022;146(5):e18-e43.

**Additional** **Table 2. Healthy Eating Index-2015 Components & Scoring Standards**

| **Component** | **Maximum points** | **Standard for maximum score** | **Standard for minimum score of zero** |
| --- | --- | --- | --- |
| ***Adequacy*** | | | |
| Total Fruits | 5 | ≥0.8 cup equiv. per 1,000 kcal | No Fruit |
| Whole Fruits | 5 | ≥0.4 cup equiv. per 1,000 kcal | No Whole Fruit |
| Total Vegetables | 5 | ≥1.1 cup equiv. per 1,000 kcal | No Vegetables |
| Greens and Beans | 5 | ≥0.2 cup equiv. per 1,000 kcal | No Dark Green Vegetables or Legumes |
| Whole Grains | 10 | ≥1.5 oz equiv. per 1,000 kcal | No Whole Grains |
| Dairy | 10 | ≥1.3 cup equiv. per 1,000 kcal | No Dairy |
| Total Protein Foods | 5 | ≥2.5 oz equiv. per 1,000 kcal | No Protein Foods |
| Seafood and Plant Proteins | 5 | ≥0.8 oz equiv. per 1,000 kcal | No Seafood or Plant Proteins |
| Fatty Acids | 10 | (PUFAs + MUFAs)/SFAs ≥2.5 | (PUFAs + MUFAs)/SFAs ≤1.2 |
| ***Moderation*** | | | |
| Refined Grains | 10 | ≤1.8 oz equiv. per 1,000 kcal | ≥4.3 oz equiv. per 1,000 kcal |
| Sodium | 10 | ≤1.1 gram per 1,000 kcal | ≥2.0 grams per 1,000 kcal |
| Added Sugars | 10 | ≤6.5% of energy | ≥26% of energy |
| Saturated Fats | 10 | ≤8% of energy | ≥16% of energy |

1. Krebs-Smith SM, Pannucci TE, Subar AF, et al. Update of the Healthy Eating Index: HEI-2015. J Acad Nutr Diet. Sep 2018;118(9):1591-1602.

**Additional** **Table 3** Sensitivity analysis of the association between the Life’s Essential 8 scores and frailty when excluding participants with cancer (n=1987).

|  | **Model 1^a^** | |  | **Model 2^b^** | |  | **Model 3^c^** | |
| --- | --- | --- | --- | --- | --- | --- | --- | --- |
|  | **OR (95% CI)** | ***P*** |  | **OR (95% CI)** | ***P*** |  | **OR (95% CI)** | ***P*** |
| **LE8 score** | | | | | | | | |
| Low | 1 (Reference) | / |  | 1 (Reference) | / |  | 1 (Reference) | / |
| Moderate | 0.40(0.22–0.73) | 0.004 |  | 0.35(0.20–0.64) | 0.001 |  | 0.44(0.25–0.77) | 0.006 |
| High | 0.06(0.02–0.23) | <0.001 |  | 0.05(0.01–0.21) | <0.001 |  | 0.10(0.03–0.35) | 0.001 |
| Per SD increase | 0.52(0.41–0.66) | <0.001 |  | 0.48(0.37–0.62) | <0.001 |  | 0.54(0.41–0.70) | <0.001 |

OR, odds ratio; SD, standard deviation; CI, confidence interval

^a^ No covariates were adjusted.

^b^ Age, sex, and race were adjusted.

^c^ Age, sex, race, education level, rate of family income to poverty, marital status were adjusted.

**Additional** **Table 4** Sensitivity analysis of the association between the Life’s Essential 8 scores and frailty when excluding participants with stroke (n=2349).

|  | **Model 1^a^** | |  | **Model 2^b^** | |  | **Model 3^c^** | |
| --- | --- | --- | --- | --- | --- | --- | --- | --- |
|  | **OR (95% CI)** | ***P*** |  | **OR (95% CI)** | ***P*** |  | **OR (95% CI)** | ***P*** |
| **LE8 score** | | | | | | | | |
| Low | 1 (Reference) | / |  | 1 (Reference) | / |  | 1 (Reference) | / |
| Moderate | 0.45(0.26–0.80) | 0.008 |  | 0.42(0.24–0.74) | 0.004 |  | 0.50(0.29–0.87) | 0.016 |
| High | 0.17(0.05–0.64) | 0.011 |  | 0.17(0.04–0.64) | 0.011 |  | 0.26(0.08–0.84) | 0.025 |
| Per SD increase | 0.58(0.45–0.75) | <0.001 |  | 0.55(0.42–0.72) | <0.001 |  | 0.61(0.47–0.79) | 0.001 |

OR, odds ratio; SD, standard deviation; CI, confidence interval.

^a^ No covariates were adjusted.

^b^ Age, sex, and race were adjusted.

^c^ Age, sex, race, education level, rate of family income to poverty, marital status were adjusted.
